# Food insecurity and postnatal depression: the mediating effect of perceived social support among women in Khayelitsha, South Africa

**DOI:** 10.1007/s00127-025-02986-1

**Published:** 2025-09-05

**Authors:** S. Mathew, C. Lund, N. Seward

**Affiliations:** 1 Department of Child and Adolescent Psychiatry, Institute of Psychiatry, Psychology and Neuroscience, King’s College London, London, UK; 2 Centre for Global Mental Health, Health Service and Population Research Department, Institute of Psychiatry, Psychology and Neuroscience, King’s College London, London, UK; 3 Department of Psychiatry and Mental Health, Alan J Flisher Centre for Public Mental Health, University of Cape Town, Building B, 46 Sawkins Road, Rondebosch, 7700 Cape Town, South Africa; 4 Centre for Clinical Brain Sciences, University of Edinburgh, Edinburgh, UK

**Keywords:** Social support, Postnatal depression, Food insecurity, Social causation, Mediation, Interventional effects

## Abstract

**Purpose:**

Understanding the mechanisms through which poverty influences perinatal depression can provide insight into how to develop interventions to improve maternal mental health. To address this question, we aim to estimate indirect effects of important mediators on the causal relationship between food insecurity and symptoms of postnatal depression.

**Methods:**

We used data from the control arm of the Africa Focus on Intervention Research for Mental health – South Africa (AFFIRM-SA) trial that included pregnant women with perinatal depression. Interventional effects (used for models that may have multiple correlated mediators) were used to decompose the total effect of food insecurity captured at baseline on symptoms of perinatal depression reducing by at least 40% (using the 17-item HDRS instrument - yes/no) at three months after delivery of the baby, into the following indirect effects: number of antenatal visits attended; suicidality at eight months gestation; and levels of social support captured at eight months gestation using the Multidimensional Scale of Perceived Social Support.

**Results:**

Food insecurity was associated with a 15% reduced probability of symptoms of depression improving at three months post-delivery (-0·151, bias-corrected 95% CI: − 0.267, -0·032), of which 48% was mediated through reduced levels of social support in women exposed to food insecurity (-0.073: bias-corrected 95% CI: -0.146, -0.029). There was no conclusive evidence to support the mediating effects of attending antenatal visits and suicidality.

**Conclusions:**

Our findings suggest that providing social support can help to reduce symptoms of postnatal depression. Future research should explore developing and evaluating a package of care for pregnant women with perinatal depression that improves food security and levels of social support. This research suggests that policy makers and practitioners have a renewed focus on increasing social support systems for women during the perinatal period, especially in cases of food insecurity.

**Supplementary Information:**

The online version contains supplementary material available at 10.1007/s00127-025-02986-1.

## Introduction

About 12% of women during the perinatal period suffer maternal depression worldwide [1]. This is significantly higher in low- and middle-income countries (LMICs), where depression prevalence is about 19.2% in the antenatal period and 18.7% in the postnatal period, compared to a prevalence in high-income countries of 9.2% and 9.5% respectively [1]. Differences in the burden of maternal depression can be explained in part by the large treatment gap in LMICs, as well as an increase in risk factors for perinatal depression such as poverty and intimate partner violence [2].

Unravelling the complexity surrounding how social determinants such as poverty influence depression is important to improving maternal mental health outcomes. There is strong evidence that poverty is associated with common mental health issues such as depression or anxiety [3, 4]. There are two possible explanations for the association between poverty and mental ill-health. The social causation hypothesis suggests that social factors such as poverty and violence increase the risk of mental illness as individuals face stress due to environmental factors [5, 6]. The social drift hypothesis on the other hand suggests that those with poor mental health drift into poverty during the course of their life. For example, mental illness may result in disability, loss of employment, and subsequently cause individuals to fall into poverty [6]. Research suggests that both social causation and social drift occur simultaneously, leading to those with poor mental health to be caught in a vicious cycle of poverty and mental illness [6].

Given the cyclic nature of poverty and mental health, the burden of poor maternal mental health has far-reaching consequences in low-resource settings. For example, research from Cape Town, South Africa suggests that mothers with depression are more likely to be unemployed, have lower household incomes, and experience catastrophic expenditure up to a year postpartum, compared to mothers without depression [7]. Breaking this cycle is critical to achieving better health, economic, and other social outcomes.

The buffering hypothesis states that social support can reduce the negative effect of psychosocial stressors on mental health. This can happen through two potential mechanisms- either by reducing the stress appraisal response itself, or by reducing the stress reaction [8]. Empirical evidence also suggests that there are some social factors that can protect against the effects of the multi-dimensional poverty on mental ill-health. For example, social support has been shown to protect against depression and other common mental disorders (CMDs) [9, 10]. A systematic review studying the impact of social support on postpartum depression in Asia found higher levels of social support reduced levels of postpartum depression [11]. Similar trends can be seen in other populations, with a meta-analysis on the predictors of postpartum depression in Ethiopia showing that poor social support was significantly associated with the risk of postpartum depression [12].

There has been some evidence indicating that poverty influences depression by reducing social support, and that social support may mediate this relationship [13, 14]. The Thinking Healthy Peer Programme (THP) was a peer led intervention that aimed to improve symptoms of perinatal depression and was evaluated in two separate trials in India and Pakistan [15, 16]. Findings from these trials suggested that THP reduced symptoms of depression. A mediation analysis using data from these trials revealed that behavioural activation and social support independently mediated the effects of the intervention on perinatal depression scores [15, 16]. Other interventions which have a focus on peer support have shown to be significantly effective against perinatal depression when compared to controls [17]. This suggests that potentially integrating social support aspects into mental health interventions could support better outcomes.

A recent systematic review assessing the relationship between food security and the risk of depression has shown that food insecurity significantly increases the likelihood of being depressed or stressed, in keeping with the relationship between poverty and depression [18]. In addition to this, cross-sectional surveys in various LMICs have shown an association between food insecurity and social support, with greater food security being associated with higher levels of social support [19, 20]. A study conducted in Uganda found that among pregnant women, the association between food insecurity and depressive symptoms was moderated by social support, i.e. the association was stronger amongst women with low social support; however this study was cross-sectional so conclusions about temporal associations are limited [21]. The current study aims to improve our understanding of the temporal associations between food insecurity, social support and depressive symptoms among perinatal women.

We aim to use a robust causal inference framework, interventional effects, to simultaneously evaluate the mediating effects of social support levels and other important mediators, on the relationship between moderate/high levels of food insecurity and symptoms of perinatal depression reducing by at least 40% using longitudinal data from the control arm of a trial that evaluated the effectiveness of a psychological intervention in reducing levels of maternal depression [22].

## Methods

### Setting

The data in this study was collected as a part of the Africa Focus on Intervention Research for Mental Health – South Africa (AFFIRM-SA) trial, an individually randomised controlled trial aimed at improving maternal depression outcomes among perinatal women in South Africa using a task sharing approach [23, 24]. Data was collected from Khayelitsha, a peri-urban settlement outside Cape Town. There is one district hospital, five community health centres and eight day clinics that serve the population of approximately 400,000 people. Families in this area face high levels of poverty and unemployment. Housing is also in poor condition, often without electricity and water. Residents also tend to live in overcrowded accommodation with poor sanitation systems [23]. Recruitment was carried out at two antenatal clinics that were part of community health centres. Details regarding recruitment and data collection strategies have been described previously [23].

#### Participants

This analysis uses data from the control arm of the AFFIRM-SA trial (*n* = 216). The eligibility criteria for the trial were that women had to be at least 18 years of age and no more than 28 weeks pregnant, able to give informed consent and speak isiXhosa, living in Khayelitsha, attending their first antenatal visit, and scoring 13 or higher on the Edinburgh Postnatal Depression Scale (EPDS). Those who required urgent medical or mental health care were excluded. Data were collected from women presenting to two antenatal clinics - Michael Mapongwana Community Health Centre (CHC) or Site B CHC - for routine antenatal check-ups. Eligible participants were randomly assigned to either the control or intervention condition.

### Measures

All measures used in this analysis were collected at three assessment visits: the first antenatal check-up which was considered baseline; eight months gestation in the antenatal period; and three months postnatal.

#### Exposure

When large percentages of a population are poor, typical measures of poverty such as income and consumption alone are not reliable [25]. This is potentially because poorer communities tend to have strong informal networks for sharing resources in cases of scarcity. In addition to this, income and consumption measures are often unreliable in an informal economy [25]. In such instances, a measure such as food insecurity would be more appropriate. We therefore selected food insecurity as our exposure of interest, captured using The Household Food Insecurity Access Scale (HFIAS) [26].

HFIAS captures levels of food insecurity through a questionnaire with answers ranging from 0 (never) to 3 (often) [26, 27]. This results in scores ranging between 0 and 27, with higher scores indicating increasing levels of insecurity. Scores are categorised into the following four levels: (1) Food Secure; (2) Mildly Food Insecure; (3) Moderately Food Insecure, and; (4) Severely Food Insecure [26, 27]. These levels were determined depending on affirmative responses to more severe conditions such as going to bed hungry or going a whole day without eating, and how often these conditions are experienced [27]. For the purposes of this study household HFIAS scores were converted into a binary variable, with households facing either no or mild food insecurity (unexposed), and households facing moderate to severe food insecurity (exposed).

#### Outcome

Our outcome was symptoms of perinatal depression reducing by at least 40% captured three months after delivery using the Hamilton Depression Rating Scale (HDRS-17) that had been adapted and validated for the AFFIRM trial [28]. The HDRS-17 consists of 17 items ranging from weight loss, insomnia, and agitation to the ability to carry out usual activities [28, 29].

#### Mediators

A mediator is defined as a variable that is on the causal pathway between the initial exposure (in our case, food insecurity) and the outcome (symptoms of postnatal depression at three months after delivery). It is therefore a variable that is causally influenced by the intervention and in turn causally influences the outcome [30]. Although our mediator of interest is levels of social support, it is also important to capture the mediating effect of other factors associated with food insecurity, levels of social support, and symptoms of postnatal depression. Failing to do so will bias the estimates from our models. Additional mediators were therefore selected if they were associated with levels social support, food insecurity, or symptoms of postnatal depression (*p* < 0.1). This relatively large *p* value was selected to ensure that all potential mediators were included to minimise the chances of severe confounding bias in the model.

The mediators for our analyses were captured at the eight-month antenatal visit, prior to the three-month postnatal follow-up visit where the outcome measure was assessed. This helps to rule out reverse causation between mediators and the outcome where there was a sufficient time lag between when the mediators were collected and the outcome of interest.

Based on these criteria, the following mediators were selected:

##### Number of antenatal visits (Mediator 1(M1))

As part of a participant’s antenatal care package, she was offered and attended scheduled antenatal care visits. We anticipated the possibility that food insecurity would influence the number of antenatal visits as those who are highly food insecure would be less likely to spend time and resources attending the health centres. We anticipated attendance to antenatal care visits could influence not only perinatal depression, but also other mediators. This would be because during antenatal visits the health of the mother and baby would be checked, information for a healthy pregnancy and delivery would be provided, leading to women feeling supported and reassured. Potentially this could lead to a reduction in symptoms of depression. To capture this effect, we created a mediator to reflect the number of antenatal sessions attended (*n* = 1–15).

##### Suicidality (M2)

We theorised that food insecurity could increase the risk of suicidal behaviours [31]. Food insecurity has previously been associated with suicidal behaviours ranging from suicidal ideation to attempts, with theories suggesting that the association is caused due to chronic stress, that it is caused by affecting social supports, and that it caused by exacerbating feelings of hopelessness and fatalism [31]. To capture the effect of food insecurity on suicidality, we used the Suicidality Module of Mini International Neuropsychiatric Interview 6.0.0 captured at eight months gestation [32]. The measure is continuous, with items having different scores, and with higher scores indicating higher levels of suicidality [32].

##### Social support (M3)

Our main hypothesis theorised that increased food insecurity may lead to a reduction in perceived social support that would increase the symptoms of postnatal depression. To capture this effect, we used the Multidimensional Scale of Perceived Social Support (MSPSS), administered at eight months gestation [33]. The MSPSS is a questionnaire with 12 items with answers ranging from Very strongly disagree (1) to Very strongly agree (7). It examines an individual’s perception of the social support they receive from friends, family, and a special person. Scores range from 0 to 84, with higher scores denoting higher levels of social support [33]. The MSPSS has shown to have good reliability (Cronbach’s Alpha: 0.85), and has demonstrated construct validity [33].

#### Dependency of mediators on one another

A by-product of the interventional indirect effects is that in addition to the mediator-specific indirect effects, there is another indirect effect via the mediators’ mutual dependence on one another [22]. This indirect effect is close to zero when mediators are conditionally independent and non-zero when mediators interact or covary in their effects on the outcome.

#### Mediator-outcome confounders

Mediator-outcome confounders can generate spurious correlations between the mediator and outcome when unadjusted for, potentially distorting these associations. We considered potential baseline characteristics that are associated with the exposure, mediators, or the outcome, as potential confounders (*p* < 0.10). The selection process for these confounders is described in the supplementary material.

### Statistical methods

#### General

To better understand how food insecurity influenced the mediators to improve symptoms of postnatal depression, we present (1) unadjusted associations between the selected mediators and food insecurity, and (2) unadjusted associations between selected mediators and symptoms of postnatal depression. This can be seen in Tables 1 and 2 respectively.

#### Mediation analysis

**Fig. 1:**
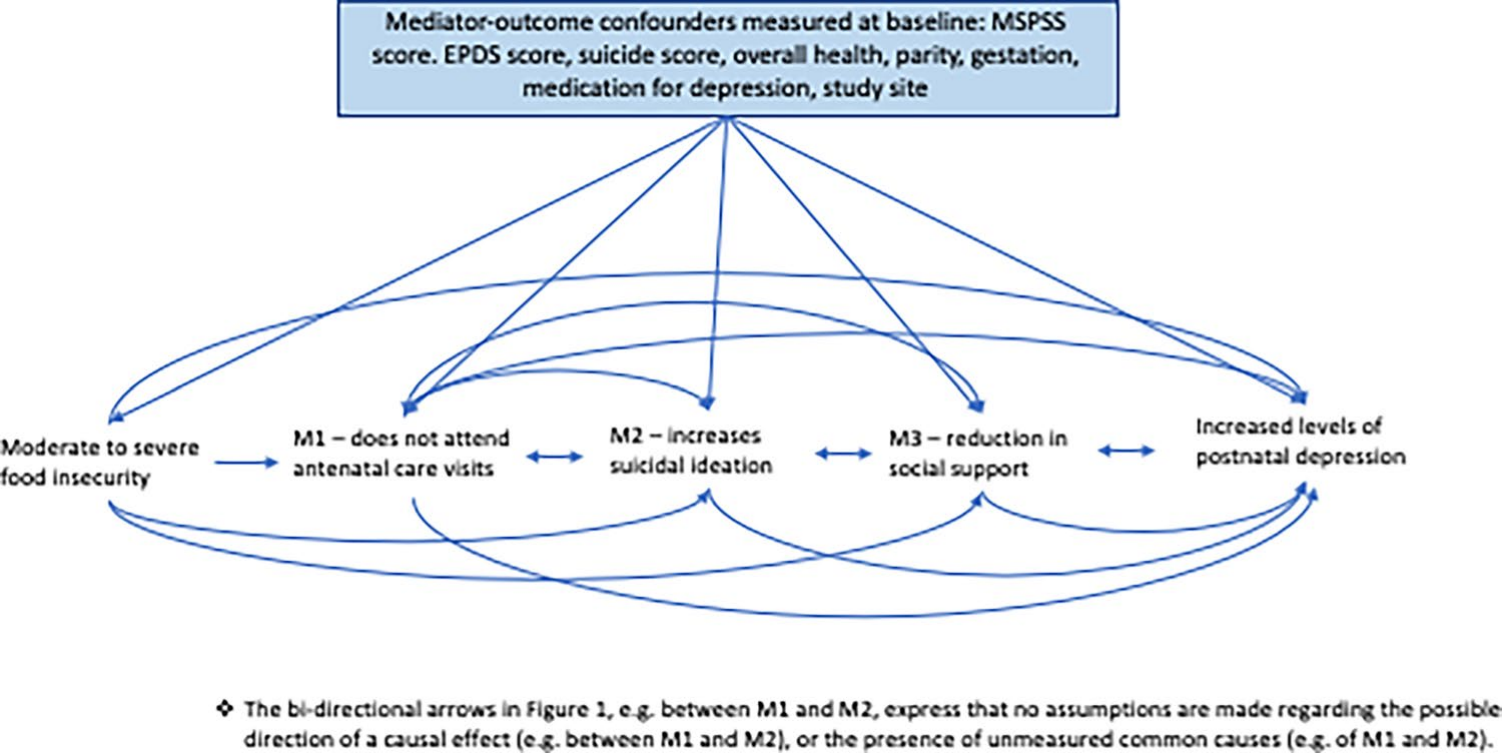
Causal model demonstrating the proposed pathways through which the exposure to moderate/severe food insecurity may lead to postnatal depression

We aimed to investigate the extent to which reduced symptoms of perinatal depression captured at three months using the HDRS questionnaire (HDRS scores improved by at least 40% between baseline and three months postnatal) were explained by the indirect effects of number of antenatal visits, suicidality levels, and levels of social support (Fig. 1). To achieve these objectives, we used the interventional (in)direct effects approach to mediation analysis to understand population-level effects relevant to this analysis [22]. Interventional effects are the latest in a series of developments that use an advanced causal inference framework to overcome limitations of other approaches to mediation such as Structural Equation Modelling [34, 35]. Findings for this analyses are described according to guidelines for reporting mediation analyses (AGReMA statement) [30]. Further details on the mediation analysis relating to the interventional effects can be found in the Supplementary Material.

##### Estimation and model fit

Estimation for the interventional indirect effects was based on Monte Carlo integration using a 1,000-fold expanded dataset [22]. The expanded dataset was created in five steps. Details of each step, and details of the models including interactions and selected confounders can be found in Supplementary Material. Bias-corrected confidence intervals were based on nonparametric bootstrap with 1,000 resamples [22].

##### Assumptions

The interventional effects have important underlying assumptions that will influence the validity of our findings if violated. The main assumptions relevant to our study is that there are no unmeasured confounders between the exposure and the mediator, and between the mediator and the outcome.

##### Sensitivity analysis

Primary outcomes for perinatal depression are reported using different instruments. We selected our outcome based on the primary outcome from the AFFIRM trial. Given different instruments capture different characteristics of perinatal depression, we also conducted the same analyses using outcomes from the Edinburgh Postnatal Depression Score. Specifically, we compared indirect effects using the outcome of HDRS scores reducing by at least 40% between baseline and three months, to that of EPDS reducing by the same percentage [36].

##### Missing data

To account for missing data, we implemented single stochastic imputation using chained equations with 10 burn-in iterations, under the assumption that data were ‘missing at random’ (MAR). Imputation models included all mediators, outcome of postnatal depression, and confounders in models to assess interventional effects. Missing variables were imputed separately for exposed (moderate to severe food insecurity) and unexposed participants (none or mild food insecurity. In each of the 1000 bootstrap samples, the imputation is done once.

## Results

Associations between Selected Mediators and Food Insecurity.

Table 1 presents findings from descriptive analysis comparing selected mediators at eight months gestation with food insecurity at baseline. Having moderate/severe food insecurity was associated with fewer antenatal visits, compared with none or mild food insecurity. Moderate to severe food insecurity was strongly associated with higher levels of suicidality compared to participants with mild or no food insecurity. Likewise, moderate to severe food insecurity was associated with lower levels of perceived social support, compared to participants with no or mild food insecurity.

**Table 1:** Comparison of mediators at eight months gestation, with exposure to moderate to severe levels of food insecurity at baseline (complete cases only)

Mediators captured at eight months gestation	No/little food insecurity^a^ *N* = 87, (54.7%)	Moderate/severe food insecurity^a^ *N* = 72, (45.3%)
M1: number of antenatal visits (mean, SD)	4.3 (2.0)	3.9 (1.5)
M2: Suicide scores^b^ (mean, SD)	1.0 (2.7)	5.4 (12.3)
M3: Social support levels^c^(mean, SD)	61.0 (11.0)	55.9 (13.0)

^a^ Levels of food insecurity captured using HFIAS questionnaire

^b^ Suicide scores captured using Suicidality Module of Mini International Neuropsychiatric Interview where higher scores indicate increased risk of suicide

^c^ Levels of social networks captured using the MSPSS questionnaire where higher scores indicate increased levels of social support

Associations between Selected Mediators and Symptoms of Perinatal Depression.

Table 2 presents a comparison between selected mediators at eight months gestation and the outcome of symptoms of perinatal depression reducing by at least 40% at three months postnatal. Women who had reduced symptoms of perinatal depression attended more antenatal visits and had higher levels of social support. Women with a higher risk of suicide, were less likely to experience a reduction in symptoms of perinatal depression.

**Table 2:** Comparison of mediators with reduction in symptoms of perinatal depression by at least 40% (complete cases only)

Mediators captured at eight months gestation	Symptoms of perinatal depression not improving at three months postnatal^a^ (*N* = 72, 47.7%)	Symptoms of perinatal depression improving at 3 months postnatal^a^ (*N* = 79, 52.3%)
M1: number of antenatal visits (mean, SD)	4.0 (1.6)	4.3 (2.0)
M2: levels of suicidality ^b^ (mean, SD)	3.8 (10.6)	2.4 (9.9)
M2: levels of social support ^c^ (mean, SD)	56.1 (12.1)	61.6 (13.8)

^a^ Perinatal depression scores captured using the adapted HDRS questionnaire

^b^ Suicide scores captured using Suicidality Module of Mini International Neuropsychiatric Interview where higher scores indicate increased risk of suicide

^c^ Levels of social networks captured using the MSPSS questionnaire where higher scores indicate increased levels of social support

### Mediation analyses

Table 3 demonstrates that at three months postnatal, women with depression and exposed to moderate/severe food insecurity at baseline, had a 15% reduced probability of symptoms of depression improving by at least 40% (adjusted difference in probability of symptoms of perinatal depression not improving by at least 40%: −0·151, bias-corrected 95% CI:- 0.268, −0·042). Of the difference in probability of symptoms of perinatal depression improving by at least 40% at three months, 48% was mediated by reduced levels of social support (mean difference in probability of symptoms of perinatal depression improving by at least 40% between those with social support levels in the exposed population, compared to women with social support levels in the unexposed population: −0.073 (95% bias-corrected confidence interval: −0.146, −0.029). This suggests that if women with moderate/high levels of food insecurity had levels of social support at similar levels to women with little or no food insecurity, they would experience a 7% increase in the probability of HDRS scores reducing by at least 40% at population level. There was no evidence to support mediation through the indirect effects of suicide scores, or though antenatal visits. Lastly, there was no evidence to support the indirect effect attributable to the mutual dependence of the mediators on one another (−0.000 [95% confidence interval: −0.004, 0.005).

**Table 3:** Total effect and interventional (in)direct effects for the causal association between moderate to severe food insecurity on reduction in HDRS scores by at least 40%

Total causal effect and interventional (in)direct effects	Difference in mean probability of HDRS scores reducing by at least 40% (bias-corrected 95% CI)^a, b, c, d^
Total effect of food insecurity on reduced symptoms of perinatal depression at three months postnatal	−0.151 (−0.268, −0.032)
Interventional direct effect	−0.088 (−0.219, 0.044)
Interventional indirect effect through attending antenatal sessions (M1)	0.006 (−0.023, 0.036)
Interventional indirect effect through an increase in suicidal score (M2)	−0.019 (−0.056, 0.015)
Interventional indirect through reduced levels of social support networks (M3)	−0.073 (−0.146, − 0.029)
Intervention effects through the dependency of mediators on one another	−0.000 (0.004, 0.005)

^a^ Estimates have been adjusted for mediator-outcome confounders of baseline HDRS scores, baseline MSPSS scores, baseline suicidality scores, number of previous pregnancies, baseline alcohol use disorders identification test score, overall health at baseline, gestation when enrolled in the trial, and study site

^b^ Estimation for the different effects was based on Monte-Carlo integration, using a 1000-fold expanded data-set

^c^ Bias-corrected confidence intervals were based on nonparametric bootstrap with 1000 resamples

^d^ Missing data has been imputed by exposure to food insecurity (unexposed and exposed) separately, using single imputation stochastic models

### Sensitivity analyses

Estimates from our analyses comparing indirect effects using a reduction of HDRS scores by at least 40% with estimates using the outcomes of EPDS scores improving by at least 40% and recovery from perinatal depression (EPDS score < 13) all suggest that reduced levels of social support mediated a decrease in the probability of reduced symptoms/recovery from perinatal depression. However, although bias-corrected 95% CI overlapped, estimates using EPDS scores reducing by 40%, found that increased suicide risk in women exposed to food insecurity, mediated a reduced probability of symptoms of perinatal depression improving.

## Discussion

Results from our robust mediation analyses suggest that the relationship between food insecurity and perinatal depression is mediated by perceived social support.

There are potential explanations for the relationship between food insecurity, perinatal depression, and self-reported levels of social support. Firstly, being from a household with high levels of food insecurity may mean there is less disposable income that is often required to take part in social activities. The inability to take part in pleasurable activities along with the absence of the protective nature of social support could then increase feelings of depression. The problem may not be limited to the lack of disposable income. For many women, leisure activities are home centred or at religious institutions like churches that may not require disposable income [37]. However, being economically disadvantaged could also mean that individuals have less leisure time, and thus have less time to be a part of social events, increasing feelings of isolation. Secondly, in communities that face relative deprivation, there tends to be a sense of community ensuring that people band together to make sure basic needs are met [38]. If depression in this group is predicated on the lack of access to basic resources, having social support could weaken or break the link between food insecurity and depression. When the community does not step in, food insecurity may be more strongly predictive of depression. Lastly, there is the well known stress buffering hypothesis of social support [8]. It is possible that when an individual faces a potentially stressful situation, in this case food insecurity, the knowledge that there are others who will provide resources to support that individual reduces the level of stress typically associated with it [8]. Social support may also help an individual reappraise a situation, for example they may provide solutions or may help build self- esteem [8]. Further research would help delineate which of these pathways may be at work. Particularly, understanding which types of social support are most effective- informational, instrumental or emotional support, will help ascertain how social support mediates the relationship between food insecurity and mental illness [39].

Antenatal visits did not significantly mediate the relationship between food insecurity and perinatal depression scores when measured using EPDS. A potential reason for this is that antenatal visits were widely accessed by the women in the sample and therefore a significant mediating effect of the visits may not be reflected in this population.

Estimates from the mediating effects of suicidality are conflicting and need to be interpreted with caution. On one hand suicidality was not a mediator when using HDRSscores. On the other hand, our findings from the sensitivity analyses using an outcome of a reduction in EPDS scores by 40% compared to a reduction in HDRS scores by 40%, indicate suicidality may also be a potential mediator. There are possible reasons for this difference including that the HDRS and EPDS capture different constructs of perinatal depression. Whereas the EPDS captures a broader range of psychological distress, including symptoms of anxiety, the HDRS only captures symptoms of depression [28, 40, 41]. A possible explanation therefore is that HDRS scores did not capture symptoms of anxiety being more sensitive to the mediating effect of suicidality, if at all present.

Findings from our analyses may not be generalisable to pregnant women in other deprived and underserved contexts. Specifically, the participants in our study were women that were already facing psychological distress, given they had scored 13 or above on the EPDS. Therefore, the results of this study may not transfer onto a population that is not already distressed. Similarly, Khayelitsha faces high levels of relative deprivation, and the trends in this study may not carry over to places where such deprivation is not as pronounced, or has different characteristics [23]. Nevertheless, findings may be generalisable to other peri-urban areas with characteristics similar to Khayelitsha, and among women who are dealing with distress in the context of food insecurity. Our findings provide valuable insight on the potential features of a package of interventions that can address social determinants of mental health and thereby improve symptoms of perinatal depression in deprived settings.

One of the main limitations of this analysis is the possibility of failing to adjust for confounders that influence the exposure and mediators, or the outcome and mediators. It is also possible that we are not capturing other mediators that influence the mediators in our analyses. These issues could potentially bias our findings. Nevertheless, we applied one of the most robust approaches to mediation available where we accounted for all available mediators and potential confounders. A limitation to our sample is our sample size (*n* = 216) which impacts on our ability to assess for multiple interactions between the different mediators. Longitudinal cohorts with larger sample sizes may provide more power to test these nuances. The self-report nature of the measures used, particularly for food insecurity is also a limitation. Participants were asked how frequent certain past experiences were, such as- “Did you or any household member eat a smaller meal than you felt you needed because there was not enough food?” making the responses subject to recall bias. Finally, the typical issues of carrying out a longitudinal study exist, e.g. attrition and inconsistency in data collection may insert bias into the findings. Steps were taken to reduce this, such as the assessment team being trained, supervised and supported by a research officer to ensure uniformity and consistency; home visits to conduct interviews, where necessary; and utilising multiple contact numbers to engage study participants at follow-up assessments [23]. The follow-up rates ranged from 80.4 to 93.5% across the different arms of the trial at three months postpartum [24].

Results from our robust mediation analyses has helped us to better understand the pathways through which recovery from perinatal depression can be improved in deprived settings. We now have a better understanding of the complex relationship between poverty and depression including the influencing factors of social support and potentially suicide risk and antenatal visits. Findings from our analyses could be used to inform the design of future interventions to help improve recovery from depression in this population, by identifying social support and food insecurity as potential targets for intervention. Further research into understanding what aspects of social support mediate the relationship can help create targeted interventions. For example, interventions that provide informational (such as information on best pregnancy practises) and emotional support to mothers in deprived settings may prove to be helpful. Even instrumental support, such as the expansion of low-cost childcare may improve depression outcomes. In low-resource areas, accepting support interventions through peers may be more easily palatable compared to highly-specialised delivery agents, and has proved useful and acceptable elsewhere [17].

## Supplementary Information

Below is the link to the electronic supplementary material.

Supplementary Material 1

## Data Availability

No datasets were generated or analysed during the current study.
