# Supplementary material for: Food insecurity and postnatal depression: the mediating effect of perceived social support among women in Khayelitsha, South Africa
Source: Soc Psychiatry Psychiatr Epidemiol. 2025 Sep 5;61(2):381–90. doi: 10.1007/s00127-025-02986-1 (PMC12948881; doi:10.1007/s00127-025-02986-1)
Supplement: Supplementary file 1 — Supplementary Material 1 [file 127_2025_2986_MOESM1_ESM.docx]

**Supplementary material**

1. **Selection of potential mediators**

Causal Directed Acyclic Graphs were used to identify all potential mediators and mediator-outcome confounders.^1^ Mediators were only included in the final model if they were associated with the exposure, another mediator, or the outcome (*p*<0·10). Using the above criteria ensured that we capture any potential mediator that is statistically influenced by the exposure, and influences either another mediator, or the improvement in depressive symptoms. Failing to include putative mediators that are concurrently confounders of mediator-outcome relations for other mediators can cause severe bias.^2^ For this reason, we elected to err on the side of caution and include mediators empirically associated with the outcome (or another mediator) to reduce the chances of confounding bias; hence the relatively large *p*-value threshold. Our selected mediators include number of antenatal visits (M1) , risk of suicide (M2) , and levels of social support (M3).

1. **Selection of mediator-outcome confounders**

Mediator-outcome confounders are variables that are influenced by the exposure, or influence the outcome or any one of the mediators (*p*<0.10) and included the following variables captured at baseline: levels of social support levels captured using the MSPSS questionnaire, baseline suicidality scores captured using scores captured from the Suicidality Module of Mini International Neuropsychiatric Interview, months gestation, number of previous pregnancies, HAM-D/EPDS scores, overall health of the woman, alcohol misuse use captured using the Alcohol use disorders identification test (AUDIT), medication to treat depression, and study site. We used a liberal threshold for *p*-values to ensure we captured all potential mediator-confounders which we hope will help to avoid introducing unmeasured confounding bias.

1. **Mediation analyses**

*Decomposition of total effect food insecurity on reduction of symptoms of perinatal depression by 40% into direct and indirect effects*

We decomposed the total effect of exposure to moderate/high levels of food insecurity on perinatal depression into interventional indirect effects via each of the three posited mediators and the direct effect via none of the mediators. The interventional indirect effect via a particular mediator (e.g., number of antenatal clinics attended) can be interpreted as the average change in the potential outcome (perinatal depression at three months) resulting from shifting the counterfactual distribution of that mediator from the exposed status (e.g., number of antenatal appointments attended for women exposed to moderate/high levels of food insecurity) to the unexposed status (number of antenatal appointments attended for women not exposed to food insecurity), while setting each of the remaining mediators to random draws from either the exposed or unexposed group, depending on the specific decomposition. In doing so, valid inferences are not contingent on strict assumptions, such as correctly specifying the causal ordering among the mediators which are unlikely to be feasible in our current setting and can lead to incorrect inferences when violated. (1)

Steps to estimation

Estimation for the interventional indirect effects was based on Monte Carlo integration using a 1,000-fold expanded dataset. (15) The expanded dataset was created in five steps. In the first step we fitted a marginal model for each mediator given exposure and other predictors. Specifically, we fitted linear regression models for each of the mediators to the observed data. Each model included a combination of predictors that were shown to be associated with the mediator of interest (*p*<0.10) that were combinations of the mediator-outcome confounders previously described. Interactions and non-linearities were explored and included if they had *p*<0.05 using Stata’s post-estimation command, *testparm*.

In the second step, we fitted conditional models for each of the relevant mediators (M2, M3) that account for the counterfactual joint distribution of the mediators. These are different from the marginal models, in that they include a joint distribution that is achieved by imputing the random, subject specific draws of other mediators in addition to the predictors. Specifically, the conditional models for M2 account for the values of M1, and conditional models for M3 account for the values of M2 and M1.

In the third step, the fitted mediator models were used to generate random, subject-specific Monte Carlo draws of each mediator (both conditional and marginal) for both the exposed and unexposed condition (the latter being the counterfactual), given their observed covariate values.

In the fourth step, we fitted a model for the outcome using a logistic regression model for reduction in HAM-D scores by at least 40%, separately in the exposed and unexposed groups, given the mediators and mediator-outcome confounders. We used a model selection criterion similar to that of the mediator models – that is, any relevant non-linearities and interactions with *p*<0·10 were included in the outcome model, using the post-estimation *testparm* command in Stata.

In the fifth step, we used the fitted outcome model to predict the potential outcomes in the expanded dataset given the random, subject-specific draws of the mediator counterfactuals from the third step. The interventional indirect effects were then calculated as the mean differences between potential outcomes under different hypothetical exposure levels that used marginal draws of the mediators. The interventional direct effect was calculated as the mean difference between potential outcomes in the exposed and unexposed populations when fixing all mediators to their counterfactual joint distribution (i.e. those calculated from the conditional models).

**2. Sensitivity analyses**

To facilitate comparability with other studies, we also conducted the same analyses using the definition of at least 40% reduction in the EDPS scores at three months compared to baseline as well as the outcome of recovery from depression (EPDS scores <13 at three months).

**Table 1: Sensitivity analyses comparing estimates from interventional indirect effects between the outcome of symptom of perinatal depression reducing by at least 40% using the Hamilton Depression Score and using the Edinburgh Perinatal Depression Score**

| **Effect** | **Reduction in HAMD scores by 40% between baseline and three months postnatal** | **Reduction in EPDS by 40% between baseline and three months postnatal** |
| --- | --- | --- |
|  | **Estimates (bias-corrected 95% CI)^a,b,c,d^** | **Estimates (bias-corrected 95% CI)^a,b,c,d^** |
| Total effect of moderate/severe levels of food insecurity on perinatal depression at three months | -0.151 (-0.268, -0.032) | -0.122 (-0.241, -0.018) |
| Direct effect | -0.088 (-0.219, 0.044) | -0.026 (-0.181, 0.095) |
| Indirect effect of attending antenatal care visits (M1) | 0.006 (-0.023, 0.036) | 0.002 (-0.033, 0.037) |
| Indirect effect of suicide score (M2) | -0.019 (-0.056, 0.015) | -0.030 (-0.068, -0.003) |
| Indirect through social support (M3) | -0.074 (-0.146, - 0.029) | -0.052 (-0.116, -0.014) |
| Interventional indirect effects through dependence of mediators on one another | 0.077 (0.025, 0.163) | -0.002 (-0.012, 003) |

^a^ Estimates have been adjusted for mediator-outcome confounders of baseline values of: EPDS or HAM-D scores, levels of social support, suicide score, medication to treat a psychological problem (yes/no), gestation (how many months pregnant at baseline), overall health, total audit score, number of previous pregnancies, age,and study site.

^b^ Estimation for the different effects was based on Monte Carlo integration using 1,000-fold expanded dataset

^c^ Bias-corrected confidence intervals were based on nonparametric bootstrap with 1000 resamples

^d^ Missing data has been imputed by exposure to food insecurity (unexposed and exposed) separately, using single imputation stochastic models

**References**

1. Loh WW, Moerkerke B, Loeys T, et al. Heterogeneous indirect effects for multiple mediators using interventional effect models. Epidemiol Methods. 2020;9(1).
